# Alveolar macrophages of GM-CSF knockout mice exhibit mixed M1 and M2 phenotypes

**DOI:** 10.1186/1471-2172-14-41

**Published:** 2013-09-17

**Authors:** Heidi Dalrymple, Barbara P Barna, Anagha Malur, Achut G Malur, Mani S Kavuru, Mary Jane Thomassen

**Affiliations:** 1 Program in Lung Cell Biology and Translational Research, Division of Pulmonary, Critical Care Medicine and Sleep Medicine, East Carolina University, Brody School of Medicine, 3E-149 Brody Medical Sciences Building, Greenville, NC 27834, USA; 2 Department of Microbiology and Immunology, East Carolina University, Greenville, NC, USA

**Keywords:** Interferon gamma, Activin A, Alveolar macrophages

## Abstract

**Background:**

Activin A is a pleiotrophic regulatory cytokine, the ablation of which is neonatal lethal. Healthy human alveolar macrophages (AMs) constitutively express activin A, but AMs of patients with pulmonary alveolar proteinosis (PAP) are deficient in activin A. PAP is an autoimmune lung disease characterized by neutralizing autoantibodies to Granulocyte-Macrophage Colony Stimulating Factor (GM-CSF). Activin A can be stimulated, however, by GM-CSF treatment of AMs *in vitro*. To further explore pulmonary activin A regulation, we examined AMs in bronchoalveolar lavage (BAL) from wild-type C57BL/6 compared to GM-CSF knockout mice which exhibit a PAP-like histopathology. Both human PAP and mouse GM-CSF knockout AMs are deficient in the transcription factor, peroxisome proliferator activated receptor gamma (PPARγ).

**Results:**

In sharp contrast to human PAP, activin A mRNA was elevated in mouse GM-CSF knockout AMs, and activin A protein was increased in BAL fluid. Investigation of potential causative factors for activin A upregulation revealed intrinsic overexpression of IFNγ, a potent inducer of the M1 macrophage phenotype, in GM-CSF knockout BAL cells. IFNγ mRNA was not elevated in PAP BAL cells. *In vitro* studies confirmed that IFNγ stimulated activin A in wild-type AMs while antibody to IFNγ reduced activin A in GM-CSF knockout AMs. Both IFNγ and Activin A were also reduced in GM-CSF knockout mice *in vivo* after intratracheal instillation of lentivirus-PPARγ compared to control lentivirus vector. Examination of other M1 markers in GM-CSF knockout mice indicated intrinsic elevation of the IFNγ-regulated gene, inducible Nitrogen Oxide Synthetase (iNOS), CCL5, and interleukin (IL)-6 compared to wild-type. The M2 markers, IL-10 and CCL2 were also intrinsically elevated.

**Conclusions:**

Data point to IFNγ as the primary upregulator of activin A in GM-CSF knockout mice which in addition, exhibit a unique mix of M1-M2 macrophage phenotypes.

## Background

Activin A, a pleiotrophic cytokine belonging to the transforming growth factor-beta (TGF-β) superfamily, is synthesized by many cell types throughout the body [1,2]. The molecular structure is a disulphide-linked, homodimeric glycoprotein composed of two inhibin βA chains. Activin A was first recognized as an endocrine factor, but is now known to be essential to developmental and repair processes, and total ablation is neonatal lethal [3]. Contrasting regulatory roles have been cited for Activin A in inflammation [4]. Human monocytes synthesize activin A upon stimulation with classical M1 macrophage activation inducers such as GM-CSF, LPS, and IFNγ [5,6]. Exposure of GM-CSF treated macrophages to anti-Activin A reduces M1 markers and enhances alternative M2 phenotype markers such as IL-10 [7]. Activin A also inhibits monocyte production of IL-1β and enhances IL-1 receptor antagonist production [8]. Interestingly, in severe asthma, activin A may be elevated in serum, and data from animal models suggests that activin A may suppress T helper 2 (Th2) mediated allergic responses [9]. Collectively these observations suggest multifunctional roles for activin A in inflammatory processes.

Maintenance of lung homeostasis is a complex process dependent upon a network of interacting cells and cytokines. GM-CSF is required for alveolar macrophage (AM) function and pulmonary homeostasis [10]. In genetically altered mice homozygous for a disrupted GM-CSF gene (GM-CSF knockout), hematopoiesis is normal but there is accumulation of excess lung surfactant [11,12]. This surfactant pathology mirrors that of human PAP, an autoimmune disease characterized by high levels of autoantibody to GM-CSF [12-14]. Aerosolized GM-CSF resolves the pulmonary pathology of GM-CSF knockout mice, thus demonstrating that surfactant homeostasis can be influenced by local administration of GM-CSF to the respiratory tract [15].

Previously we reported that healthy human AMs synthesize activin A in response to GM-CSF but AMs of patients with PAP are deficient in activin A [16]. In addition, PAP AMs are deficient in the nuclear transcription factor, Peroxisome Proliferator-activated Receptor, (PPARγ), a regulator of lipid and glucose metabolism that is restored by GM-CSF treatment [17]. PPARγ has also been shown to be a negative regulator of inflammation [18,19]. Interestingly, alveolar macrophages of GM-CSF knockout mice are also deficient in PPARγ [20]. The role of activin A in the lung has not been established. Because of the phenotypic similarities between human PAP and the GM-CSF knockout mouse, this study was undertaken to investigate activin A regulation in the lung. Initially, it was hypothesized that activin A might be impaired in GM-CSF knockout mice based upon previous data from PAP studies [16].

## Results

### Activin A and IFNγ are intrinsically elevated in GM-CSF knockout mice as compared to wild-type mice

**Figure 1:**
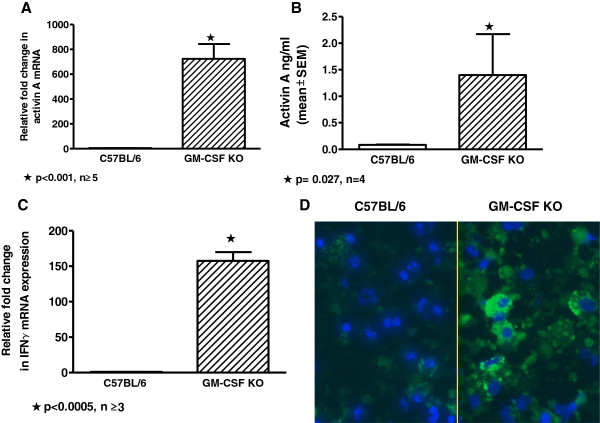
**Activin A and IFNγ are intrinsically elevated in GM-CSF knockout lung compared to wild-type mice.** Expression of mRNA is shown as a relative fold change as calculated from qPCR. **(A)** Activin A mRNA expression of BAL cells. **(B)** Levels of activin A protein in BAL fluids were quantified by ELISA. **(C)** IFNγ mRNA expression of BAL cells. **(D)** Cytospin preparations of wild-type C57Bl/6 and GM-CSF knockout BAL cells were stained with anti-IFNγ (green) and DAPI (blue), a nuclear counterstain. Elevated IFNγ protein can be seen in GM-CSF knockout BAL cells (right) compared to wild-type (left).

Unlike previous findings of activin A deficiency in human PAP [16], activin A mRNA expression of BAL cells was significantly (p <0.005) elevated in GM-CSF knockout mice compared to wild-type controls (Figure 1A). Quantification of activin A protein in BAL fluids confirmed mRNA findings with significantly (p < 0.05) elevated protein levels in GM-CSF knockout compared to wild-type (Figure 1B). GM-CSF knockout expression of follistatin, an inhibitor of activin A [21], was similar to wild-type mice (data not shown) and thus could not account for the striking elevation of activin A.

Intrinsic factors that could potentially affect activin A levels were subsequently investigated in GM-CSF knockout mice. Macrophage colony-stimulating factor (M-CSF) has been reported to be upregulated in GM-CSF knockout mice [22]. Examination of M-CSF in the current study, however, indicated no effect on activin A *in vitro* in either wild-type or GM-CSF knockout AMs (data not shown). Elevated IFNγ has been reported in lungs of GM-CSF knockout mice [23] therefore intrinsic levels of IFNγ were examined. IFNγ mRNA expression was significantly (p < 0.005) elevated in GM-CSF knockout BAL cells compared to wild-type controls (Figure 1C). Immunocytochemistry of GM-CSF knockout BAL cells confirmed mRNA results and indicated markedly increased expression of intracellular IFNγ protein compared to wild-type (Figure 1D).

### IFNγ is not elevated in human PAP BAL cells

**Figure 2:**
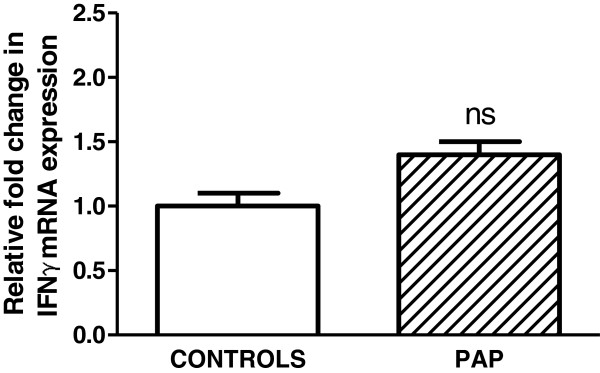
**Expression of IFNγ is not detectable in BAL cells from human PAP patients.** Expression of IFNγ mRNA in PAP patients (n = 6) did not significantly differ from that of healthy controls (n = 5).

In contrast to results from GM-CSF knockout mice, examination of IFNγ expression in human BAL cells from PAP patients revealed no significant increase compared to healthy controls (Figure 2).

### Activin A levels are enhanced by IFNγ and reduced by IFNγ blockade

**Figure 3:**
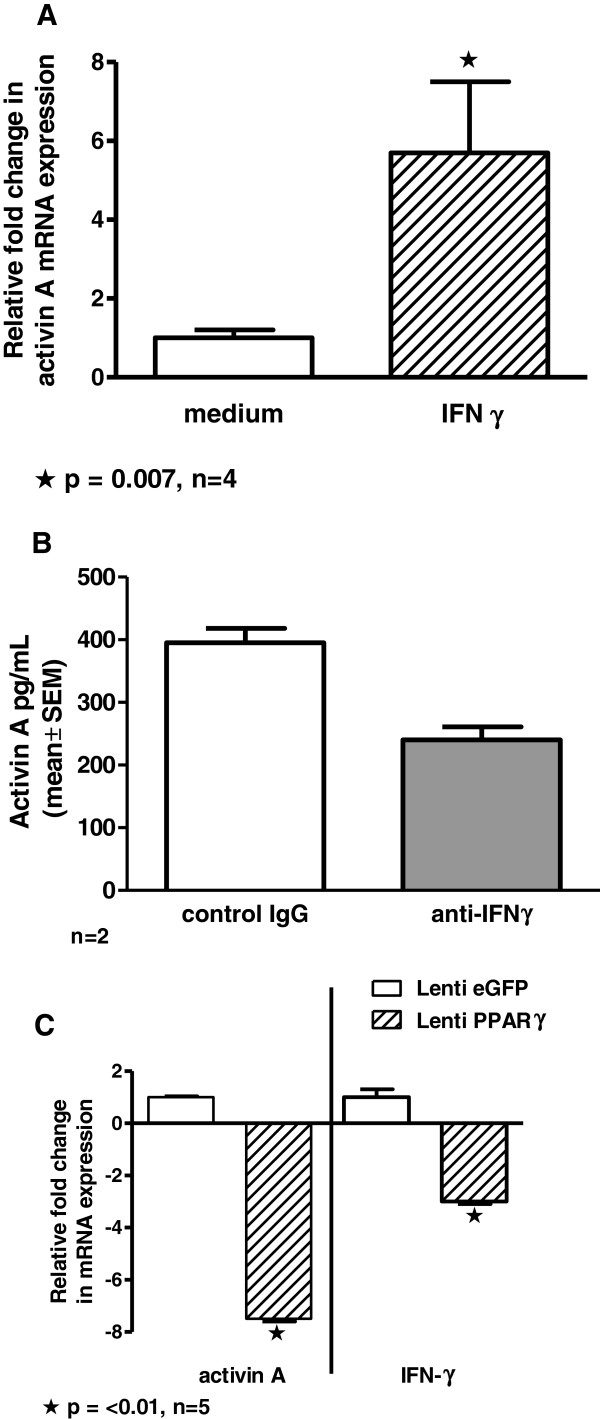
**Activin A levels are increased by IFNγ and reduced by IFNγ blockade. (A)** IFNγ upregulates activin A mRNA in wild-type alveolar macrophages cultured for 24 hours with IFNγ (100 U/ml) [n = 4]. **(B)** Antibody to IFNγ represses intrinsic activin A synthesis in GM-CSF knockout alveolar macrophages. GM-CSF knockout alveolar macrophages were cultured with irrelevant IgG or anti-IFNγ for 24 hours and activin A was determined in conditioned media by ELISA (n = 2). **(C)** BAL cells from GM-CSF knockout mice receiving intratracheal instillation of lentivirus-PPARγ or control lentivirus-EGFP were analyzed for IFNγ and activin A mRNA expression at 10 days post-transduction (n = 5).

IFNγ has been shown to upregulate activin A expression in human monocytes [5] but AMs have not been studied. Results from 24-hour *in vitro* cultures of wild-type AMs indicated that IFNγ (100 U/ml) significantly (p < 0.05) increased activin A expression (Figure 3A). To determine whether blockade of IFNγ with specific anti-IFNγ antibody would alter intrinsic activin A expression, unstimulated GM-CSF knockout AMs were cultured *in vitro* for 24 hours with irrelevant immunoglobulin (Ig) or anti-IFNγ. ELISA analysis of conditioned media indicated that anti-IFNγ reduced activin A protein synthesis compared to irrelevant Ig (Figure 3B) confirming that IFNγ blockade reduced intrinsic activin A production.

Because activin A is intrinsically elevated in PPARγ deficient GM-CSF knockout mice but severely decreased in PPARγ deficient human PAP patients [16], it appeared unlikely that PPARγ would exert a direct effect on activin A. Observations made elsewhere [24] also found no evidence of a PPARγ effect on activin A. We have shown, however, that IFNγ is elevated in macrophage-specific PPARγ knockout mice and significantly reduced after *in vivo* restoration of PPARγ via a lentivirus vector [25]. We utilized this approach to determine whether PPARγ restoration in GM-CSF knockout mice might reduce IFNγ and thereby reduce activin A. Results supported this action. Ten days post intratracheal inoculation of lentivirus reagents into GM-CSF knockout mice, BAL cell mRNA expression of both IFNγ and activin A was significantly reduced in animals receiving lentivirus-PPARγ compared to controls receiving lentivirus-eGFP (p < 0.05) (Figure 3C).

### Human alveolar macrophage activin A is increased by IFNγ

**Figure 4:**
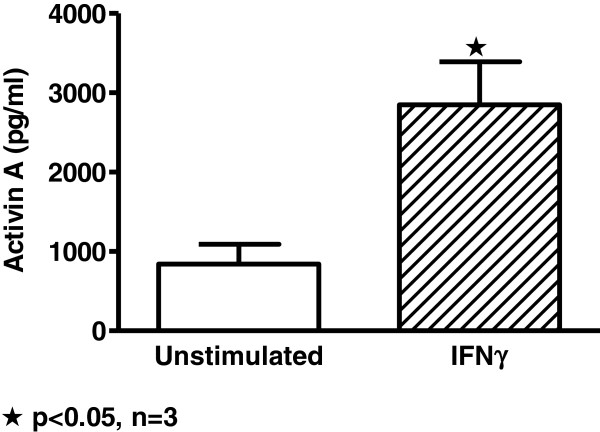
**Human alveolar macrophages from healthy donors produce activin A in response to IFNγ.** Activin A protein is increased in conditioned media from human alveolar macrophages cultured with IFNγ (1000 U/ml) for 24 hours *in vitro*.

While the above studies clearly defined IFNγ-mediated regulation of activin A in murine alveolar macrophages, it was necessary to confirm this pathway in human alveolar macrophages. *In vitro* studies demonstrated that IFNγ significantly enhanced activin A protein production (Figure 4) in healthy human alveolar macrophages. Thus activin A synthesis in both human and murine alveolar macrophages is responsive to IFNγ upregulation even though intrinsic activin A levels differ between human and mouse.

### GM-CSF BAL cells show intrinsic elevation of both M1 and M2 macrophage phenotypic markers

**Figure 5:**
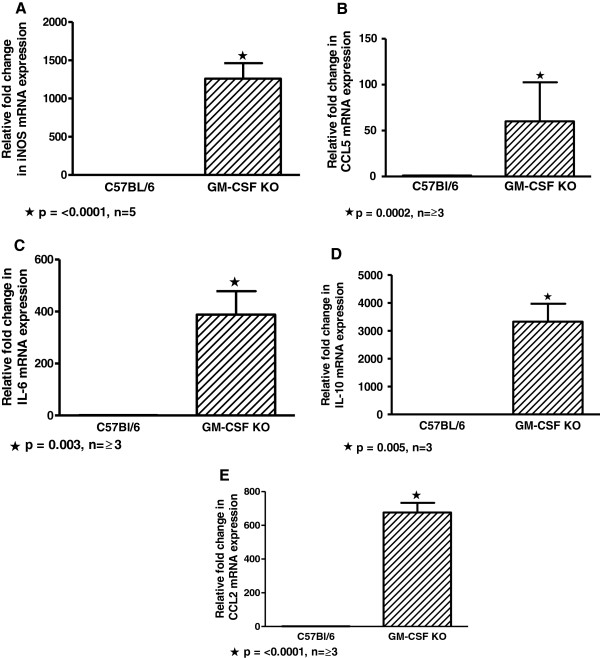
**mRNA expression of both M1 and M2 macrophage phenotypic markers is intrinsically elevated in GM-CSF knockout BAL cells compared to wild-type mice.** Elevated mRNA expression of M1 markers: **(A)** iNOS; **(B)** CCL5, and **(C)** IL-6. Elevated mRNA expression of M2 markers: **(D)** IL-10, and **(E)** CCL2.

We and others reported previously that M-CSF gene expression and protein, a cytokine associated with the M2 macrophage phenotype, was elevated in GM-CSF knockout mice [22,26]. Current data indicate that the M1-associated cytokine, IFNγ (protein and gene expression) is also increased in these mice. Therefore, it was unclear whether GM-CSF knockout BAL cells would express predominantly M1 or M2 profiles. To address this issue, we determined mRNA expression of several M1 and M2 markers in GM-CSF knockout BAL cells. With respect to M1 markers, we examined the IFNγ-regulated target gene, iNOS (Figure 5A), together with CCL5 (Figure 5B), and IL-6 (Figure 5C), and found that all were significantly elevated compared to wild-type mice. The M2 marker, IL-10, has been reported to be suppressed by elevated activin A [7,27], and in PAP, activin A deficiency is accompanied by elevated IL-10 [16,28,29]. Surprisingly, analysis of IL-10 expression in GM-CSF knockout BAL cells revealed significantly elevated levels compared to wild-type mice (Figure 5D). Analysis of another M2-associated marker, CCL2, also indicated significant elevation compared to wild-type mice (Figure 5E). These results suggested that GM-CSF knockout alveolar macrophages might constitute a mixed population of both M1 and M2 phenotypes.

## Discussion

The current findings suggest that IFNγ is a major contributory factor to the intrinsic elevation of activin A in AMs. Findings also point out a striking difference in activin A expression in human PAP and GM-CSF knockout mice despite common deficiencies of GM-CSF and PPARγ (summarized in Table 1). In parallel with activin A, GM-CSF knockout mice displayed over-expression of IFNγ [23], a positive regulator of activin A [5]. In contrast, BAL cells of PAP patients do not exhibit elevated IFNγ and activin A is deficient [16].

**Table 1:** Summary: comparison of macrophage activation regulatory factors in human pulmonary alveolar proteinosis (PAP) patients and GM-CSF knockout mice

**Regulatory factors**	**Associated macrophage phenotype**	**PAP patients**	**GM-CSF knockout mice**
**GM-CSF**	**M1**	Deficient protein, not mRNA [28]	Absent [11]
**Activin A**	**M1**	Deficient [16]	Elevated
**IL-6**	**M1**	Not done	Elevated
**CCL5**	**M1**	Not done	Elevated
**IFNγ**	**M1**	mRNA - not elevated (comparable to healthy controls)	Elevated [23]
**INOS**	**M1**	Undetectable in human alveolar macrophages (unpublished observation)	Elevated
**M-CSF**	**M2**	Elevated [30]	Elevated [22]
**PPARγ**	**M2**	Deficient [31]	Deficient [31]
**CCL2**	**M2**	Elevated [32]	Elevated
**IL-10**	**M2**	Elevated [29]	Elevated
**MMP2**	**M2**	Elevated [33]	Elevated [33]

Elevated IFNγ has been reported previously in the BAL fluids of GM-CSF knockout mice [23]. Our previous studies also found elevated IFNγ expression in macrophage-specific PPARγ knockout mice [25]. Restoration of PPARγ via lentivirus vector in these mice greatly diminished IFNγ expression [25]. In the current study, similar results were seen after PPARγ–lentivirus treatment of GM-CSF knockout mice. Such findings suggest that the PPARγ deficiency present in GM-CSF knockout mice may contribute to elevated IFNγ. GM-CSF has been shown to be a critical upregulator of PPARγ [31,34]. The total lack of GM-CSF in knockout mice may maintain an extreme PPARγ deficiency which is ineffective at repressing inflammatory mediators such as IFNγ. In human PAP, IFNγ levels are not increased despite PPARγ deficiency, furthermore, GM-CSF is not totally absent [29]. The primary etiology of PAP is considered to be an autoimmune response to GM-CSF in the form of high levels of circulating, neutralizing autoantibody to GM-CSF [13]. It is also possible that additional regulatory mechanisms are present in human lung to help prevent IFNγ buildup in PAP.

The varying characteristics of activated macrophages have led to attempts to categorize activation phenotypes [35-39]. The M1 phenotype is characterized by production of microbial or IFNγ-triggered molecules such as iNOS and IL-12. GM-CSF has been cited as an inducer of M1 phenotypes while M-CSF has been shown to induce the M2 alternative activation phenotype in which IL-10 or TGFβ may be produced [7,40]. We have shown that M-CSF is elevated in GM-CSF knockout mice [22] and in human PAP [33] which might suggest the presence of an M2 macrophage phenotype (see Table 1). Interestingly, PPARγ, which is deficient in GM-CSF knockout mice, is also a major driver of the M2 phenotype [41]. It has been pointed out however, that macrophage phenotypes were defined by carefully controlled *in vitro* conditions which may be vastly different from the *in vivo* milieu [42]. Thus the juxtaposition of both IFNγ and M-CSF in the lungs of GM-CSF knockout mice could produce the novel combination of macrophage activation phenotypes illustrated by elevated M1 (iNOS, CCL5, IL-6) and M2 (IL-10, CCL2) markers (Table 1). Other IFNγ-inducible pro-inflammatory mediators (chemokines CXCL9, CXCL10, and CXCL11) have been noted in the lungs of GM-CSF knockout mice [23]. Previously, we found that MMP-2, a matrix metalloproteinase associated with M-CSF and alternative M2 activation, is also elevated in GM-CSF knockout BAL cells [33].

## Conclusions

The current findings extend our previous studies examining pulmonary mechanisms operative in human PAP and the GM-CSF knockout mouse. It is clear that pathways of activin A regulation may utilize GM-CSF or IFNγ as stimulatory factors. In the GM-CSF knockout mouse, lack of GM-CSF may restrict production of sufficient PPARγ to control inflammation. The persistent elevation of both M-CSF and IFNγ may influence AMs to express characteristics of both M1 and M2 phenotypes. The current data emphasize the plasticity of alveolar macrophages in assuming a unique activation phenotype when regulatory pathways become dysfunctional.

## Methods

### Mice

Animal studies were conducted in conformity with Public Health Service (PHS) Policy on humane care and use of laboratory animals and were approved by the institutional animal care committee. The GM-CSF knockout mice were generated by Dr. Glenn Dranoff and have been previously described [11]. Controls consisted of C57BL/6 wild type mice obtained from Jackson Laboratory (Bar Harbor, ME). BAL cells and fluids were obtained from 8-12 week-old GM-CSF knockout mice and age and gender matched wild-type C57BL/6 controls as previously described [43]. Briefly, cytospins of BAL cells were stained with a modified Wright-Giemsa stain for differentials. A minimum of 100 cells was scored for each lavage. Mean (± SEM) BAL cells from C57BL/6 mice were composed of 98 ± 1% macrophages and 2 ± 1% lymphocytes; GM-CSF knockout BAL cells were composed of 91 ± 2% macrophages and 5 ± 1% lymphocytes. For *in vitro* studies, BAL cells were plated at 150,000 cells/well in 48-well plates as previously described [25]. Recombinant murine IFNγ was obtained from R&D Systems. Neutralizing anti-IFNγ and control antibodies were purchased from BD Biosciences. For all experiments a minimum of 3 sets of pooled BAL cells from 3-5 mice were used except where indicated.

### Human subjects

The protocol was approved by the East Carolina University Institutional Review Board and written informed consent was obtained from all patients and control subjects. Healthy control subjects had no history of lung disease and were not on medication. PAP subjects were recruited from patients undergoing routine clinical evaluation. The diagnosis of idiopathic PAP was confirmed by histopathological examination of material from open lung or transbronchial biopsies as previously described [29]. Alveolar macrophages were derived from bronchoalveolar lavage (BAL) obtained by fiberoptic bronchoscopy as previously described [29]. Differential cell counts were obtained from cytospins stained with a modified Wright’s stain. For PAP patients, the mean BAL cell percentages (means ± SEMs) were: alveolar macrophages, 83 ± 9%, and lymphocytes, 10 ± 5%. Healthy control values were: alveolar macrophages, 93 ± 2% and lymphocytes, 7 ± 2%. For *in vitro* culture, BAL cells were plated into 24-well plates (300,000 alveolar macrophages per well) or chamber slides (60,000 cells/well) as previously described [16].

### RNA purification and analysis

Total RNA was extracted from BAL cells or cultured alveolar macrophages and analyzed by Q-PCR as previously described [25]. RNA specimens were analyzed in duplicate using primer-probe sets for activin A, IL-10, iNOS, CCL2, CCL5, IL-6, IFNγ and GAPDH as previously described [25]. Data were normalized to GAPDH and expressed as fold change in mRNA expression compared to controls values as previously described [44].

### Lentivirus plasmid and transduction

The self-inactivating lentivirus expression vector used here has been described previously [45]. Construction of the lentivirus-PPARγ (lenti-PPARγ) and control lentivirus construct has also been described in detail [20,25]. Control consisted of a lentivirus vector expressing Enhanced Green Fluorescent Protein (eGFP) (lenti-EGFP). Animals received 50 ug of lentivirus vector in 50 μl PBS or PBS alone (sham) by intratracheal instillation. After 10 days, five animals per group were lavaged, BAL differential counts were obtained and RNA was extracted.

### Activin A and follistatin protein assays

Activin A or follistatin proteins (pg/ml) in BAL fluids or conditioned media from cultured alveolar macrophages were quantified by ELISA according to the manufacturer’s instructions (Serotec, Raleigh, NC; R&D Systems, Minneapolis, MN).

### Immunocytochemistry

Immunocytochemistry for IFNγ was carried out on cytospin samples from freshly isolated BAL cells using rat anti-mouse IFNγ (Santa Cruz Biotechnology,1:100) followed by goat anti-rat IgG (Invitrogen) as described [25]. Slides were counter-stained with DAPI (Invitrogen) to allow nuclear localization.

### Statistics

Data were analyzed by student’s t-test using Prism software (GraphPad). Values from treated cells were compared to untreated. Significance was defined as p ≤ 0.05.

## Abbreviations

AM: Alveolar macrophage; GM-CSF: Granulocyte macrophage colony stimulating factor; PAP: Pulmonary alveolar proteinosis; BAL: Bronchoalveolar lavage; PPARγ: Peroxisome proliferator activated receptor; IFNγ: Interferonγ; iNOS: Inducible nitric oxide synthetase.

## Competing interests

The authors declare that they have no competing interests.

## Authors’ contributions

HD contributed to acquisition of the data, analysis and interpretation of data, drafting of the manuscript and final approval of the version to be published; BPB contributed to the design, analysis and interpretation of data, drafting of the manuscript and final approval of the version to be published; AM contributed to the conception and design, acquisition of the data, analysis and interpretation of data and final approval of the version to be published; AGM contributed to acquisition of the data and final approval of the version to be published; MSK contributed to the acquisition of data and final approval of the version to be published; MJT contributed to the conception and design, acquisition of data, analysis and interpretation of data, drafting of the manuscript and final approval of the version to be published. All authors read and approved the final manuscript.

## References

[B1] Vale W Rivier J Vaughan J McClintock R Corrigan A Woo W Purification and characterization of an FSH releasing protein from porcine ovarian follicular fluid Nature 1986 321 776 779 10.1038/321776a0 3012369

[B2] Ling N Ying SY Ueno N Shimasaki S Esch F Hotta M Pituitary FSH is released by a heterodimer of the beta-subunits from the two forms of inhibin Nature 1986 321 779 782 10.1038/321779a0 3086749

[B3] Matzuk MM Kumar TR Vassalli A Bickenbach JR Roop DR Jaenisch R Functional analysis of activins during mammalian development Nature 1995 374 354 356 10.1038/374354a0 7885473

[B4] De Kretser DM O’Hehir RE Hardy CL Hedger MP The roles of activin A and its binding protein, follistatin, in inflammation and tissue repair Mol Cell Endocrinol 2012 359 101 106 10.1016/j.mce.2011.10.009 22037168

[B5] Shao L Frigon NL Jr Sehy DW Yu AL Lofgren J Schwall R Regulation of production of activin A in human marrow stromal cells and monocytes Exp Hematol 1992 20 1235 1242 1426103

[B6] Abe M Shintani Y Eto Y Harada K Kosaka M Matsumoto T Potent induction of activin A secretion from monocytes and bone marrow stromal fibroblasts by cognate interaction with activated T cells J Leukoc Biol 2002 72 347 352 12149426

[B7] Lacey DC Achuthan A Fleetwood AJ Dinh H Roiniotis J Scholz GM Defining GM-CSF-and macrophage-CSF-dependent macrophage responses by in vitro models J Immunol 2012 188 5752 5765 10.4049/jimmunol.1103426 22547697

[B8] Ohguchi M Yamato K Ishihara Y Koide M Ueda N Okahashi N Activin A regulates the production of mature interleukin-1beta and interleukin-1 receptor antagonist in human monocytic cells J Interferon Cytokine Res 1998 18 491 498 10.1089/jir.1998.18.491 9712365

[B9] Karagiannidis C Hense G Martin C Epstein M Ruckert B Mantel PY Activin A is an acute allergen-responsive cytokine and provides a link to TGF-beta-mediated airway remodeling in asthma J Allergy Clin Immunol 2006 117 111 118 10.1016/j.jaci.2005.09.017 16387593

[B10] Trapnell BC Whitsett JA GM-CSF regulates pulmonary surfactant homeostasis and alveolar macrophage-mediated innate host defense Annu Rev Physiol 2002 64 775 802 10.1146/annurev.physiol.64.090601.113847 11826288

[B11] Dranoff G Crawford AD Sadelain M Ream B Rashid A Bronson RT Involvement of granulocyte-macrophage colony-stimulating factor in pulmonary homeostasis Science 1994 264 713 716 10.1126/science.8171324 8171324

[B12] Dranoff G Mulligan RC Activities of granulocyte-macrophage colony-stimulating factor revealed by gene transfer and gene knockout studies Stem Cells 1994 12 Suppl 1 173 182 7696961

[B13] Kitamura T Tanaka N Watanabe J Uchida K Kanegasaki S Yamada Y Idiopathic pulmonary alveolar proteinosis as an autoimmune disease with neutralizing antibody against granulocyte/macrophage colony-stimulating factor J Exp Med 1999 190 875 880 10.1084/jem.190.6.875 10499925 PMC2195627

[B14] Trapnell BC Whitsett JA Nakata K Pulmonary alveolar proteinosis N Engl J Med 2003 349 2527 2539 10.1056/NEJMra023226 14695413

[B15] Reed JA Ikegami M Cianciolo ER Lu W Cho PS Hull W Aerosolized GM-CSF ameliorates pulmonary alveolar proteinosis in GM-CSF-deficient mice Am J Physiol 1999 276 L556 L563 10198353 10.1152/ajplung.1999.276.4.L556

[B16] Bonfield TL Barna BP John N Malur A Culver DA Kavuru MS Suppression of activin A in autoimmune lung disease associated with anti-GM-CSF J Autoimmun 2006 26 37 41 10.1016/j.jaut.2005.10.004 16337108

[B17] Bonfield TL Raychaudhuri B Malur A Abraham S Trapnell BC Kavuru MS PU.1 regulation of human alveolar macrophage differentiation requires granulocyte-macrophage colony-stimulating factor Am J Physiol Lung Cell Mol Physiol 2003 285 L1132 L1136 12896880 10.1152/ajplung.00216.2003

[B18] Pascual G Fong AL Ogawa S Gamliel A Li AC Perissi V A SUMOylation-dependent pathway mediates transrepression of inflammatory response genes by PPAR-[gamma] Nature 2005 437 759 763 10.1038/nature03988 16127449 PMC1464798

[B19] Glass CK Saijo K Nuclear receptor transrepression pathways that regulate inflammation in macrophages and T cells Nat Rev Immunol 2010 10 365 376 10.1038/nri2748 20414208

[B20] Thomassen MJ Barna BP Malur A Bonfield TL Farver CF Malur A ABCG1 is deficient in alveolar macrophages of GM-CSF knock-out mice and patients with pulmonary alveolar proteinsosis J Lipid Res 2007 48 2762 2768 10.1194/jlr.P700022-JLR200 17848583

[B21] Mather JP Follistatins and alpha 2-macroglobulin are soluble binding proteins for inhibin and activin Horm Res 1996 45 207 210 10.1159/000184789 8964585

[B22] Shibata Y Berclaz YP Chroneos ZC Yoshida M Whitsett JA Trapnell BC GM-CSF regulates alveolar macrophage differentiation and innate immunity in the lung through PU.1 Immunity 2001 15 557 567 10.1016/S1074-7613(01)00218-7 11672538

[B23] Su YC Rolph MS Hansbro NG Mackay CR Sewell WA Granulocyte-macrophage colony-stimulating factor is required for bronchial eosinophilia in a murine model of allergic airway inflammation J Immunol 2008 180 2600 2607 18250471 10.4049/jimmunol.180.4.2600

[B24] Weigert J Neumeier M Wanninger J Schober F Sporrer D Weber M Adiponectin upregulates monocytic activin A but systemic levels are not altered in obesity or type 2 diabetes Cytokine 2009 45 86 91 10.1016/j.cyto.2008.10.017 19128983

[B25] Malur A Mccoy AJ Arce S Barna BP Kavuru MS Malur AG Deletion of PPARγ in alveolar macrophages is associated with a Th-1 pulmonary inflammatory response J Immunol 2009 182 5816 5822 10.4049/jimmunol.0803504 19380830

[B26] Bonfield TL Thomassen MJ Farver CF Abraham S Koloze MT Zhang X Peroxisome proliferator-activated receptor-{gamma} regulates the expression of alveolar macrophage macrophage colony-stimulating factor J Immunol 2008 181 235 242 18566389 10.4049/jimmunol.181.1.235 PMC2819287

[B27] Sierra-Filardi E Puig-Kroger A Blanco FJ Nieto C Bragado R Palomero MI Activin A skews macrophage polarization by promoting a proinflammatory phenotype and inhibiting the acquisition of anti-inflammatory macrophage markers Blood 2011 117 5092 5101 10.1182/blood-2010-09-306993 21389328

[B28] Thomassen MJ Raychaudhuri B Bonfield TL Malur A Abraham S Barna BP Elevated IL-10 inhibits GM-CSF synthesis in pulmonary alveolar proteinosis Autoimmunity 2003 36 285 290 10.1080/0891693031000152688 14567558

[B29] Thomassen MJ Yi T Raychaudhuri B Malur A Kavuru MS Pulmonary alveolar proteinosis is a disease of decreased availability of GM-CSF rather than an intrinsic cellular defect Clin Immunol 2000 95 85 92 10.1006/clim.2000.4859 10779401

[B30] Bonfield TL Russell D Burgess S Malur A Kavuru MS Thomassen MJ Autoantibodies against granulocyte macrophage colony-stimulating factor are diagnostic for pulmonary alveolar proteinosis Am J Respir Cell Mol Biol 2002 27 481 486 10.1165/rcmb.2002-0023OC 12356582

[B31] Bonfield TL Farver CF Barna BP Malur A Abraham S Raychaudhuri B Peroxisome proliferator-activated receptor-gamma is deficient in alveolar macrophages from patients with alveolar proteinosis Am J Respir Cell Mol Biol 2003 29 677 682 10.1165/rcmb.2003-0148OC 12805087

[B32] Bonfield TL John N Malur A Barna BP Culver DA Kavuru MS Elevated monocyte chemotactic proteins 1, 2, and 3 in pulmonary alveolar proteinosis are associated with chemokine receptor suppression Clin Immunol 2005 114 79 85 10.1016/j.clim.2004.09.004 15596412

[B33] Bonfield TL Swaisgood CM Barna BP Kavuru MS Thomassen MJ Elevated gelatinous activity in pulmonary alveolar proteinosis: role of macrophage-colony stimulating factor J Leukoc Biol 2006 79 133 139 16275889 10.1189/jlb.0805447

[B34] Ricote M Huang JT Welch JS Glass CK The peroxisome proliferator-activated receptor-γ (PPARγ) as a regulator of monocyte/macrophage function J Leukoc Biol 1999 66 733 739 10577502 10.1002/jlb.66.5.733

[B35] Mosser DM Edwards JP Exploring the full spectrum of macrophage activation Nat Rev Immunol 2008 8 958 969 10.1038/nri2448 19029990 PMC2724991

[B36] Gordon S Martinez FO Alternative activation of macrophages: mechanism and functions Immunity 2010 32 593 604 10.1016/j.immuni.2010.05.007 20510870

[B37] Liu G Yang H Modulation of macrophage activation and programming in immunity J Cell Physiol 2013 228 502 512 10.1002/jcp.24157 22777800

[B38] Gordon S Alternative activation of macrophages Nat Rev Immunol 2003 3 23 35 10.1038/nri978 12511873

[B39] Sica A Mantovani A Macrophage plasticity and polarization: in vivo veritas J Clin Invest 2012 122 787 795 10.1172/JCI59643 22378047 PMC3287223

[B40] Fleetwood AJ Lawrence T Hamilton JA Cook AD Granulocyte-macrophage colony-stimulating factor (CSF) and macrophage CSF-dependent macrophage phenotypes display differences in cytokine profiles and transcription factor activities: implications for CSF blockade in inflammation J Immunol 2007 178 5245 5252 17404308 10.4049/jimmunol.178.8.5245

[B41] Chawla A Control of macrophage activation and function by PPARs Circ Res 2010 106 1559 1569 10.1161/CIRCRESAHA.110.216523 20508200 PMC2897247

[B42] Weidenbusch M Anders HJ Tissue microenvironments define and get reinforced by macrophage phenotypes in homeostasis or during inflammation, repair and fibrosis J Innate Immun 2012 4 463 477 10.1159/000336717 22507825 PMC6741480

[B43] Malur A Baker AD McCoy AJ Wells G Barna BP Kavuru MS Restoration of PPARgamma reverses lipid accumulation in alveolar macrophages of GM-CSF knockout mice Am J Physiol Lung Cell Mol Physiol 2011 300 L73 L80 10.1152/ajplung.00128.2010 21036914

[B44] Livak KJ Schmittgen TD Analysis of relative gene expression data using real-time quantitative PCR and the 2(−Delta Delta C(T)) Method Methods 2001 25 402 408 10.1006/meth.2001.1262 11846609

[B45] Malur AG Chattopadhyay S Maitra RK Banerjee AK Inhibition of STAT 1 phosphorylation by human parainfluenza virus type 3 C protein J Virol 2005 79 7877 7882 10.1128/JVI.79.12.7877-7882.2005 15919942 PMC1143680

